# Density functional theory study of Mobius boron-carbon-nitride as potential CH_4_, H_2_S, NH_3_, COCl_2_ and CH_3_OH gas sensor

**DOI:** 10.1098/rsos.220778

**Published:** 2022-11-02

**Authors:** Mohammad Tanvir Ahmed, Shariful Islam, Farid Ahmed

**Affiliations:** Department of Physics, Jahangirnagar University, Dhaka 1342, Bangladesh

**Keywords:** Mobius strip, gas sensor, DFT, boron-carbon-nitride, adsorption

## Abstract

The interesting properties of Mobius structure and boron-carbon-nitride (BCN) inspired this research to study different characteristics of Mobius BCN (MBCN) nanoribbon. The structural stability and vibrational, electrical and optical properties are analysed using the density functional theory. The gas-sensing ability of the modelled MBCN structure was also studied for methane, hydrogen sulfide, ammonia, phosgene and methanol gases. The negative adsorption energy and alteration of electronic bandgap verified that MBCN is very sensitive toward the selected gases. The complex structures showed a high absorption coefficient with strong chemical potential and 7 ps–0.3 ms recovery time. The negative change in entropy signifies that all the complex structures were thermodynamically stable. Among the selected gases, the MBCN showed the strongest interaction with methanol gas.

## 1. Introduction

The emission of many dangerous and poisonous gases into the environment from diverse sources has grown dramatically in recent years. Many poisonous gases occur in the environment, including carbon monoxide (CO), methane (CH_4_), sulfur dioxide (SO_2_), carbon dioxide (CO_2_), nitric oxide (NO), ammonia (NH_3_), methanol (CH_3_OH), hydrogen sulfide (H_2_S) and phosgene (COCl_2_), which are created by motorized traffic, power plants, industry, biological waste and so on [1–4]. Though CH_4_ is not a toxic gas, it is extremely flammable and can cause pulmonary toxicity due to excessive inhalation [3]. H_2_S gas is a colourless, acidic and combustible toxic gas that can have severe and irreversible effects on the nervous system [5]. NH_3_, COCl_2_ and CH_3_OH are also highly toxic gases produced via different industrial applications [1,2]. Monitoring these dangerous gases is critical in ensuring a healthier living environment [4,6], which inspired researchers to develop novel ways of sensing these chemicals. Following the momentous discovery of graphene, researchers have shown a strong interest in two-dimensional materials.

**Figure 1.**
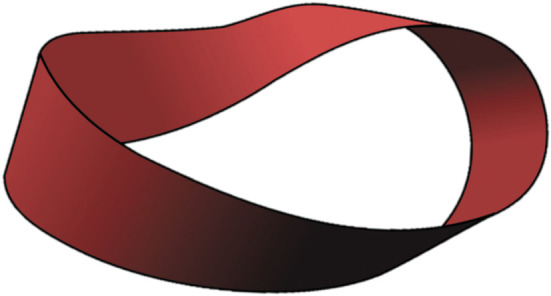
Mobius strip.

The famous illustration of a one-sided surface is the Mobius strip (figure 1), which is a three-dimensional structure made by twisting one end of a rectangular strip of plastic or paper through 180 degrees and then uniting the ends [7]. Ajami *et al*. [8] were the first to synthesize a stable Mobius aromatic hydrocarbon in 2003. Caetano *et al*. [9] in 2008 reported that the highest occupied molecular orbital (HOMO)–lowest unoccupied molecular orbital (LUMO) transition energy increases with the number of twists in the Mobius strip of graphene nanoribbon. Wang *et al*. [10] theoretically investigated graphene Mobius strip and observed better stability for different widths. Zhang *et al*. [11] experimentally synthesized Mobius graphene via the self-assembly method, which showed improved conductivity, carrier concentration and mobility. The Mobius strip of graphene nanoribbon also showed suitability as a topological insulator with better magnetic properties [12]. Chung & Chai [13] studied the electronic properties of a Mobius strip of fused benzene rings using density functional theory (DFT) and reported that the number of benzene rings is intimately related to the active orbital at the molecular edge. In 2022, Segawa *et al*. [14] successfully synthesized Mobius carbon nanobelts with a green-blueish fluorescence. Graphene-like hexagonal boron-nitride has become a new potential material for gas sensing among researchers. Experimentally synthesized boron-nitride nanosheet (BNNS) showed high sensitivity towards CH_4_ molecules [15]. Lin *et al*. [16] reported an experimental synthesis of hexagonal BNNS, which showed better NH_3_- and ethanol-sensing properties. The strong thermal conductivity and temperature stability of hexagonal BN make it a potential gas-sensing material, especially in severe conditions. The adsorption of various gas molecules (NO_2_, NO, NH_3_, CO, CH_4_, H_2_ and others) on the BN surface has been studied experimentally and computationally [17]. Theoretical research on hexagonal BNNS by Yu *et al.* revealed the highly selective adsorption of four flavonoids from bee honey (i.e. apigenin, kaempferol, myricetin and quercetin). The study found that the adsorbed flavonoids could be recovered using ethanol as an elution solvent [18].

Two-dimensional boron-carbon-nitride (BCN) nanosheet with a high surface-to-volume ratio is also a potential material for gas-sensing applications and has shown better sensitivity towards PH_3_, SO_2_, HCN and CO gases. Mawwa *et al*. [19] developed in-plane graphene-like BNNS theoretically and studied the gas sensitivity towards CO and SO_2_ molecules, which showed greater adsorption energy for CO adsorption. Wei *et al*. [20] reported a theoretical study on penta-BCN monolayer sheet, which showed a strong affinity towards NH_3_, NO, CO and H_2_S gases. Azevedo *et al*. [21] theoretically investigated zigzag and armchair BN Mobius stripes, revealing that the addition of a carbon defect causes an insulator to semiconductor transition.

Being a material with interesting structural and electrical properties, we chose the Mobius structure to sense hazardous gases. Here, we modelled a Mobius BCN (MBCN) structure and studied its structural, optical and electrical properties. We also studied the sensing ability of MBCN for CH_4_, H_2_S, NH_3_, COCl_2_ and CH_3_OH gases.

## 2. Theoretical details

The quantum mechanical approach, DFT, was used to investigate the structural, chemical, electrical, thermodynamic and other properties of the suggested structure in order to determine its appropriateness as a gas sensor. We chose the B3LYP, CAMB3LYP, HSEH1PBE and B3PW91 functionals with 6–31G(d) basis set to optimize the geometry of the pristine structure, among which B3LYP revealed the minimum total energy. Hence, the rest of the calculations of all the structures are performed by B3LYP functional. The optical properties and vibrational modes are obtained through energy and frequency calculation, respectively, with the same functional. All the simulations were performed by Gaussian 09W (Revision D.01-SMP) in closed shell formalism [19]. The adsorption energy was calculated from the following equation [19]:

2.1 $$E_{\mathrm{Ads}}=E_{\mathrm{MBCN}+\mathrm{Gas}}-E_{\mathrm{MBCN}}-E_{\mathrm{Gas}},$$

where *E*_MBCN + Gas_, *E*_MBCN_ and *E*_Gas_ denote the total energy of the MBCN with the adsorbed gas molecules, pristine MBCN and gas molecules, respectively. When orbitals are estimated by the expansion of analytic basis functions, the basis set superposition error (*E*_BSSE_) arises in the electronic structure of molecules [22], which needs to be analysed. Using the counterpoise method to estimate the energy corresponds to BSSE, which later can provide the corrected adsorption energy (*E*_Ads, Corr_) from the relation [19]

2.2 $$E_{\mathrm{Ads},\;\mathrm{Corr}}=E_{\mathrm{Ads}}+E_{\mathrm{BSSE}}.$$

A quantum mechanical system's minimum possible energy is zero-point energy (ZPE). All quantum mechanical systems experience fluctuations even in the ground state and have a ZPE. The ZPE correction is also significantly important in calculating the adsorption properties. The zero-point corrected adsorption energy (*E*_Ads,ZPE_) was calculated by the following equation:

2.3 $$E_{\mathrm{Ads},\;\mathrm{ZPE}}=E_{\left(\mathrm{MBCN}+\mathrm{gas},\mathrm{ZPE}\right)}-E_{\left(\mathrm{MBCN},\mathrm{ZPE}\right)}-E_{\left(\mathrm{gas},\mathrm{ZPE}\right)},$$

where *E*_(MBCN + gas, ZPE)_, *E*_(MBCN, ZPE)_ and *E*_(gas, ZPE)_ are the zero-point corrected energy of the MBCN + gas complex structure, pristine MBCN and gas molecules, respectively [23].

The B3LYP method cannot describe the dispersion among molecules to provide higher accuracy of adsorption [24]; hence we incorporated the dispersion-corrected B3LYP-D3 calculation to obtain the dispersion-corrected adsorption energy (*E*_Ads, Disp_) from the following equation:

2.4 $$E_{\mathrm{Ads},\;\mathrm{Disp}}=E_{\left(\mathrm{MBCN}+\mathrm{gas},\mathrm{Disp}\right)}-E_{\left(\mathrm{MBCN},\mathrm{Disp}\right)}-E_{\left(\mathrm{gas},\mathrm{Disp}\right)},$$

where *E*_(MBCN + gas, Disp)_, *E*_(MBCN, Disp)_ and *E*_(gas, Disp)_ are the dispersion-corrected energy of the MBCN + gas complex structure, pristine MBCN and gas molecules, respectively [19,24,25].

The electronic properties of the complexes were obtained from the density of states (DOS), energy gap and charge transfer between the atoms of MBCN and the gases, calculated by Hirshfeld charge (HC) analysis and Mulliken charge (MC) analysis and electrostatic potential (ESP) map. The energy gap was calculated from the HOMO and the LUMO energies through the equation

2.5 $$E_{g}=E_{\mathrm{LUMO}}-E_{\mathrm{HOMO}},$$

where *E*_LUMO_ and *E*_HOMO_ are the LUMO and HOMO energies, respectively [19].

To learn more about the reactivity and chemical stability of conjugated structures, we looked at quantum mechanical descriptors (QMD) such as chemical potential (*μ*), global hardness (*η*), global softness (*δ*), electronegativity (*χ*) and electrophilicity (*ω*), which were estimated by the following sets of equations [26]:

2.6 $$\eta =\frac{(E_{\mathrm{LUMO}}-E_{\mathrm{HOMO}})\;}{2},$$

2.7 $$\mu =\frac{(E_{\mathrm{LUMO}}+E_{\mathrm{HOMO}})\;}{2},$$

2.8 $$\delta =\frac{1}{\eta },$$

2.9 $$\omega =\frac{\mu^{2}}{2\eta }$$

and

2.10 χ= −μ.

Furthermore, because of being an essential consideration in the field of gas sensing, the recovery time of the complexes was estimated. We investigated thermodynamic metrics such as changes in enthalpy (Δ*H*), entropy (Δ*S*) and Gibbs free energy (Δ*G*) throughout the adsorption process to determine the thermal stability of pristine and conjugated nanostructures. The parameters Δ*H* and Δ*G* were calculated as follows [27]:

2.11 $$\Delta \phi =\phi_{\mathrm{MBCN}+\mathrm{Gas}}-\phi_{\mathrm{MBCN}}-\phi_{\mathrm{Gas}},$$

where *ϕ* stands for enthalpy as well as Gibbs free energy. *ϕ*_MBCN + Gas_, *ϕ*_MBCN_ and *ϕ*_Gas_ are enthalpy or Gibbs free energy of the complex structures, pristine MBCN and gas molecules, respectively. The change in entropy was calculated as [27] follows:

2.12 $$\Delta S=\frac{\Delta H-\Delta G}{T},$$

where *T* denotes the temperature.

## 3. Results and discussion

### 3.1. Geometric structures

**Figure 2.**
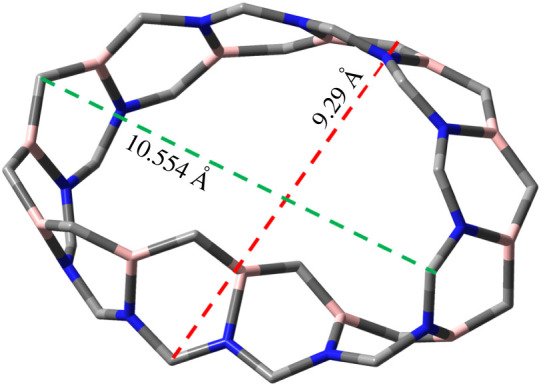
MBCN (without boundary hydrogen).

**Figure 3.**
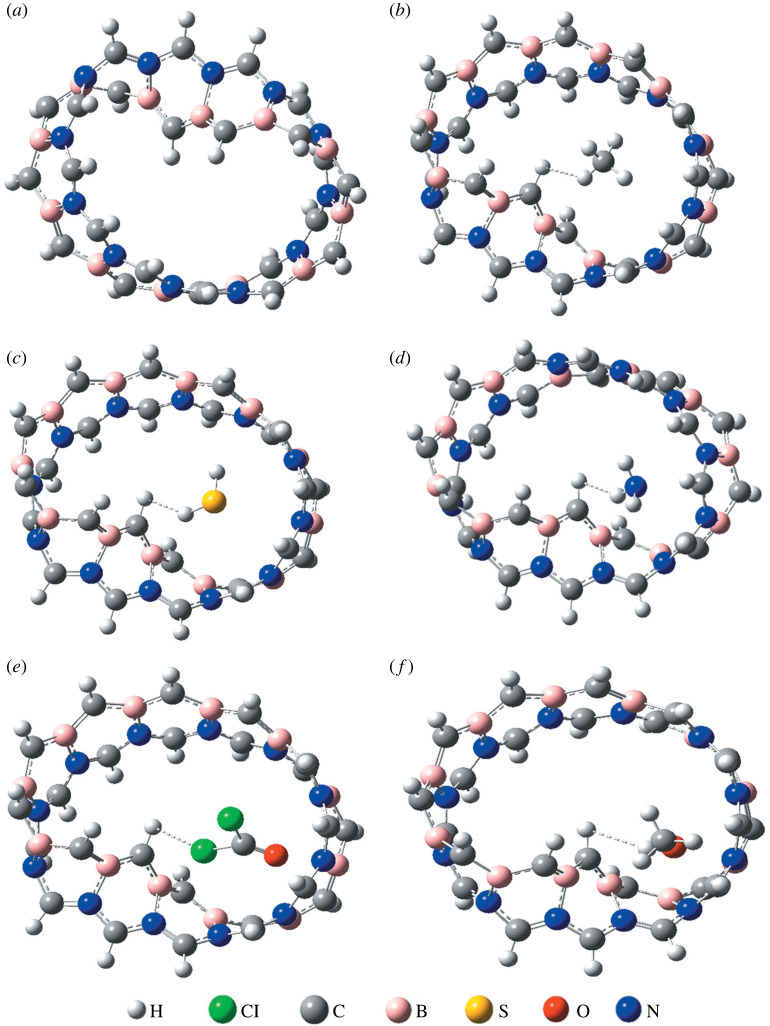
Geometrical structures of (*a*) MBCN, (*b*) MBCN + CH_4_, (*c*) MBCN + H_2_S, (*d*) MBCN + NH_3_, (*e*) MBCN + COCl_2_ and (*f*) MBCN + CH_3_OH.

We modelled Mobius nanoribbon of different carbon, boron and nitrogen concentration, e.g. C_48_, B_2_C_44_N_2_, B_4_C_40_N_4_, B_8_C_32_N_8_ and B_12_C_24_N_12_ possessing total electronic energy of −50 157 eV, −50 343 eV, −50 529.3 eV, −50 927.2 eV and −51 270.2 eV, respectively. The minimum total energy is possessed by B_12_N_12_C_24_ revealing the total energy decreases with the reduction of carbon concentration. The B_12_C_24_N_12_ structure is more energetically stable compared to Mobius graphene and hence we studied the B_12_C_24_N_12_ MBCN structure in our study. The average diameter of the structure is about 9.92 Å (figure 2). The boundary valances are completed by introducing hydrogen bonds. The CH_4_, H_2_S, NH_3_, COCl_2_ and CH_3_OH gas-sensing properties of the modelled MBCN structure have been studied. Figure 3 shows the pristine MBCN and the complex structures of MBCN with the adsorbed gas. The variation in structural properties can be identified from the change in bond lengths. The bond lengths of the gas molecules are shown in table 1, whereas the average bond lengths of the pristine and complex MBCN structures are shown in table 2.

**Table 1.** Bond lengths (Å) of the gas molecules.

bond type	CH_4_	H_2_S	NH_3_	COCl_2_	CH_3_OH
C–H	1.094				1.096
S–H		1.349			
N–H			1.001		
C=O				1.181	
C–Cl				1.765	
O–H					0.969
C–O					1.41

**Table 2.** Average bond lengths (Å) of the MBCN and MBCN + gas complexes.

bond type	MBCN	MBCN + CH_4_		MBCN + H_2_S		MBCN + NH_3_		MBCN + COCl_2_		MBCN + CH_3_OH	
		MBCN	CH_4_	MBCN	H_2_S	MBCN	NH_3_	MBCN	COCl_2_	MBCN	CH_3_OH
B–C	1.495	1.496		1.5		1.495		1.497		1.494	
B–N	1.56	1.557		1.567		1.573		1.561		1.561	
N–C	1.379	1.369		1.365		1.371		1.363		1.364	
C–H			1.093								1.096
S–H					1.356						
N–H							1.022				
C=O									1.189		
C–Cl									1.756		
O–H											0.981
C–O											1.422

The B–N bond length of the pristine MBCN is slightly larger than that obtained in nanosheets, but B–C and C–N bond lengths satisfy the previous results [19,28]. This is because the bending of the BCN ribbon slightly widens the B–N distance. It is observed that the bond lengths of MBCN structure are very slightly varied after gas adsorption, which signifies the structural deformation of MBCN is very much less. The structural deformation of the gas molecules is also observed to be very much less after adsorption.

The adsorption energy, BSSE corrected adsorption energy, ZPE adsorption energy and dispersion-corrected adsorption energy are calculated from equations (2.1)–(2.4), respectively, and listed in Table 3 along with the adsorption distance (AD). All adsorption energies are negative which verifies that the interaction between MBCN and the toxic gases is attractive. Based on the adsorption energy, it can be inferred that CH_3_OH shows more interaction with MBCN compared to other gases. From the AD, it is seen that methanol is adsorbed very closely to the pristine structure, whereas CH_4_ and COCl_2_ are more distant from the adsorbent.

**Table 3.** Adsorption energy, AD and recovery time.

structures	*E*_Ads_ (eV)	*E*_Ads, corr_ (eV)	*E*_Ads,ZPE_ (eV)	*E*_Ads,Disp_ (eV)	AD (Å)	recovery time
MBCN + CH_4_	−0.05	−0.04	−0.016	−0.33	3.0	7.0 ps
MBCN + H_2_S	−0.23	−0.17	−0.166	−0.56	2.43	7.3 ns
MBCN + NH_3_	−0.47	−0.46	−0.40	−0.65	2.46	0.3 ms
MBCN + COCl_2_	−0.19	−0.1	−0.167	−0.77	3.25	1.6 ns
MBCN + CH_3_OH	−0.53	−0.36	−0.474	−0.83	2.29	8.0 ms

The recovery time is one of the essential properties of gas-sensing materials and can be calculated from the following equation [19]:

3.1 $$\tau =\frac{1}{\;f_{o}}e^{-E_{\mathrm{Ads}}/KT},$$

where *T* and *K* denote the temperature and the Boltzmann constant (8.617 × 10^−5^ eV K^−1^), respectively, and *f*_o_ is the used frequency. A sensor can be recovered experimentally by exposing it to vacuum UV radiation with a frequency of (10^12^ to 3 × 10^14^ Hz) and a temperature of 298–350 K. In this study, we estimated the recovery time using *f*_o_ = 10^12^ Hz and *T* = 298 K [19]. The recovery times of the complexes are listed in table 3. From equation (3.1), it is observed that higher adsorption energy corresponds to higher recovery time and vice versa. The maximum recovery time is obtained to be 8.0 ms for MBCN + CH_3_OH complex due to higher adsorption energy. On the other hand, the MBCN + CH_4_ complex possesses the least recovery time (approx. 7 ps) due to very much lower adsorption energy. MBCN showed comparatively lower adsorption energies than neutral WO_3_ nanosheet towards CH_4_, H_2_S, and NH_3_ gases, which leads to a fast recovery time of MBCN compared to WO_3_ [29]. The adsorption energy toward CH_3_OH gas is much higher for MBCN compared to Fe-MoS_2_ [30], graphyne [31], graphene, BNNS and BCN [32]. MBCN showed stronger adsorption of COCl_2_ gas compared to B_12_P_12_ [33] and B_12_N_12_ [34] nanocage sensors.

### 3.2. Vibrational modes

**Figure 4.**
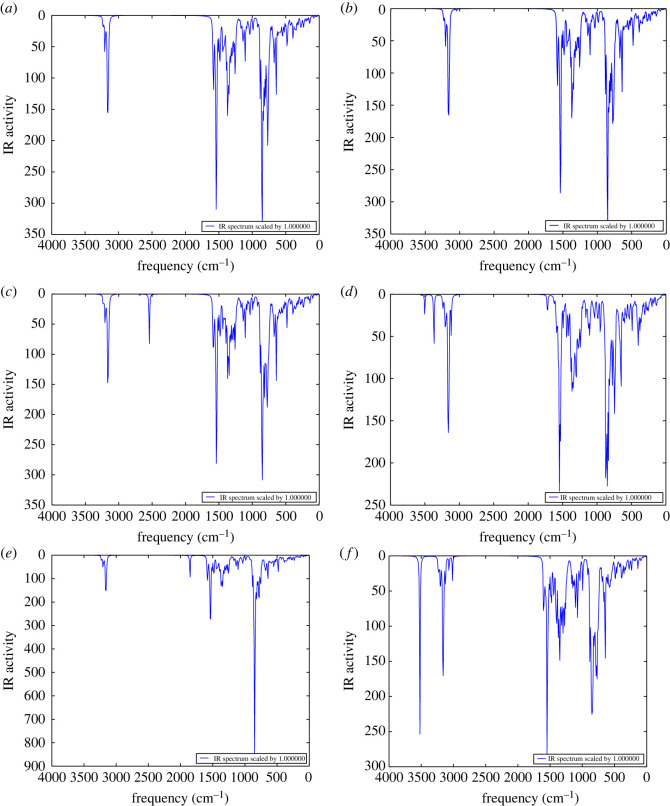
IR spectra of (*a*) MBCN, (*b*) MBCN + CH_4_, (*c*) MBCN + H_2_S, (*d*) MBCN + NH_3_, (*e*) MBCN + COCl_2_ and (*f*) MBCN + CH_3_OH.

The natural existence of the MBCN structure is confirmed via frequency calculation, which reveals all real frequency values. The vibrations of all the structures range from 7 cm^−1^ to 3500 cm^−1^ (figure 4). Among them, the C–H stretching with the boundary hydrogens is observed between 3194 and 3224 cm^−1^. 1536–1543 cm^−1^ and 1296–1308 cm^−1^ wavenumbers represent the C–N stretching and B–C stretching vibrations, respectively. B–N vibrations are observed at low wavenumbers ranging from 598 cm^−1^ to 624 cm^−1^. For the MBCN + H_2_S complex, the peak ranging from 2542 cm^−1^ to 2684 cm^−1^ represents the S–H stretching of the H_2_S molecule. For the MBCN + NH_3_ complex, the N–H stretching vibrations are identified by the peaks at 3550 cm^−1^ and 3502 cm^−1^. The strong peak at 3520 cm^−1^ in the MBCN + CH_3_OH complex represents O–H stretching. The 1854 cm^−1^ peak represents the C=O stretching in the MBCN + COCl_2_ complex. Minor alterations of peak positions were observed after CH_4_ adsorption due to less interaction between CH_4_ and MBCN.

### 3.3. Electronic properties

Tables 4 and 5 show the average HCs of the elements before and after adsorption, respectively. All the elements in the MBCN structure show a very slight change in average HCs, signifying a minor displacement of charge after gas adsorption. The gas molecules also showed significant charge displacement after adsorption. In the MBCN structure, the B atoms are partially positively charged, whereas the N and C atoms are partially negatively charged due to their high electronegativity, i.e. bond pair electrons (BPE) are attracted toward the N and C atoms; similarly, BPE is displaced further from the B atoms.

**Table 4.** Average Hirshfield charges of the elements before adsorption.

elements	MBCN	CH_4_	H_2_S	NH_3_	COCl_2_	CH_3_OH
C	−0.076	−0.152			0.248	−0.021
B	0.110					
S			−0.134			
N	−0.072			−0.353		
O					−0.187	−0.264
Cl					−0.03	
H	0.057	0.0381	0.0672	0.118		0.071

**Table 5.** Average Hirshfield charges of the elements in the complexes.

elements	MBCN + CH_4_		MBCN + H_2_S		MBCN + NH_3_		MBCN + COCl_2_		MBCN + CH_3_OH	
	MBCN	CH_4_	MBCN	H_2_S	MBCN	NH_3_	MBCN	COCl_2_	MBCN	CH_3_OH
C	−0.075	−0.157	−0.076		−0.077		−0.076	0.241	−0.076	−0.021
B	0.111		0.110		0.109		0.11		0.111	
N	−0.071		−0.071		−0.069	−0.295	−0.072		−0.071	
H	0.057	0.034	0.056	0.068	0.056	0.109	0.056		0.057	0.064
S				−0.152						
O								−0.199		−0.256
Cl								−0.012		

**Figure 5.**
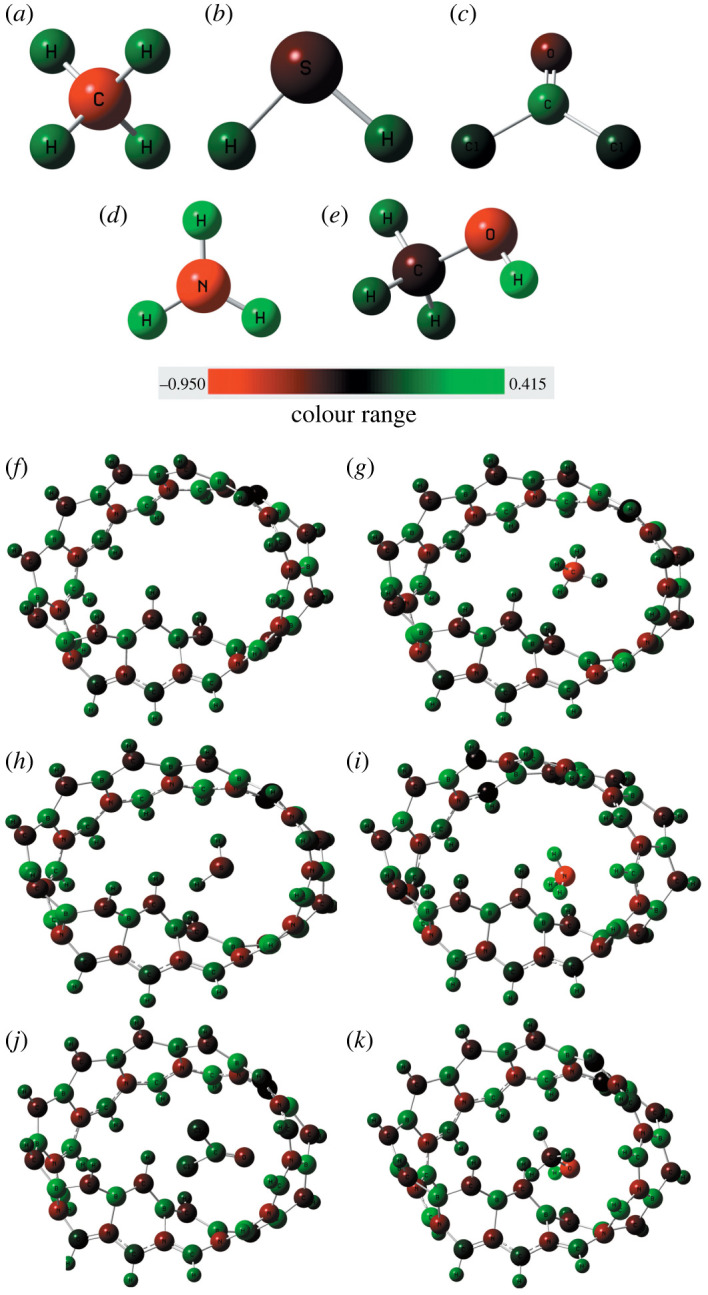
MC distribution of (*a*) CH_4_, (*b*) H_2_S, (*c*) NH_3_, (*d*) COCl_2_, (*e*) CH_3_OH, (*f*) MBCN, (*g*) MBCN + CH_4_, (*h*) MBCN + H_2_S, (*i*) MBCN + NH_3_, (*j*) MBCN + COCl_2_ and (*k*) MBCN + CH_3_OH structures.

The MCs of the elements are shown in figure 5. The green colour represents a more positive partial charge, the red colour represents a negative partial charge and the dark colour represents almost neutrality. The MC distribution also satisfies the HC analysis. The B atoms show positive partial charges, whereas N and C atoms show negative partial charges as studied in the HC distribution. A very slight change in MCs is observed after the adsorption process.

**Figure 6.**
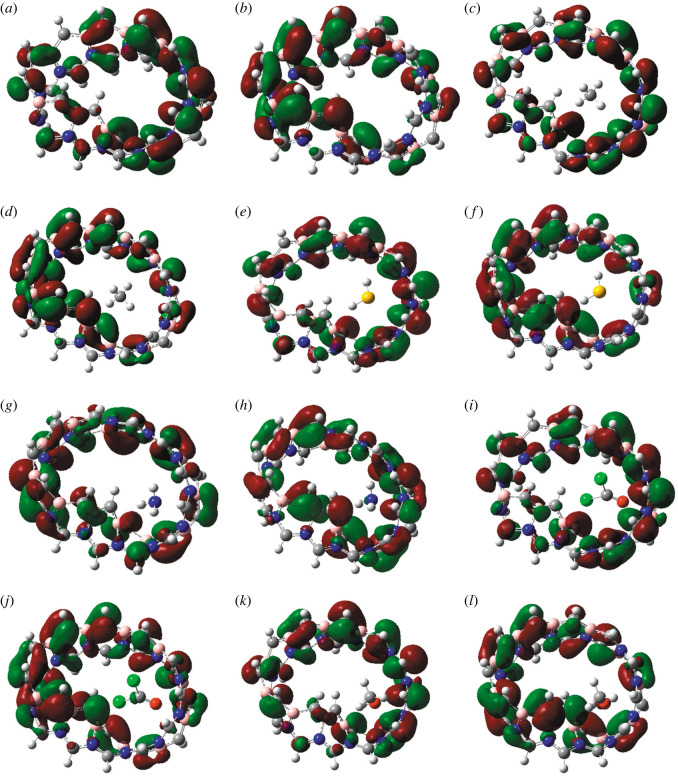
HOMOs of (*a*) MBCN, (*c*) MBCN + CH_4_, (*e*) MBCN + H_2_S, (*g*) MBCN + NH_3_, (*i*) MBCN + COCl_2_ and (*k*) MBCN + CH_3_OH complexes; LUMOs of (*b*) MBCN, (*d*) MBCN + CH_4_, (*f*) MBCN + H_2_S, (*h*) MBCN + NH_3_, (*j*) MBCN + COCl_2_ and (*l*) MBCN + CH_3_OH complexes.

Figure 6 shows the HOMO and LUMO diagrams of the pristine and gas-adsorbed MBCN. Slight distinctions are observed in the HOMOs and LUMOs after gas adsorption. The LUMO energy slightly increased (became more negative) after the adsorption of the gases except for NH_3_. The LUMOs of MBCN, MBCN + CH_4_, MBCN + H_2_S, MBCN + NH_3_, MBCN + COCl_2_ and MBCN + CH_3_OH are located at −3.028 eV, −3.0419 eV, −3.1176 eV, −2.932 eV, −3.0702 eV and −3.104 eV, whereas the HOMOs are located at −3.967 eV, −3.9725 eV, −4.002 eV, −3.951 eV, −3.959 eV and −3.91 eV, respectively. In every complex, HOMO and LUMO are localized in the MBCN structure.

**Figure 7.**
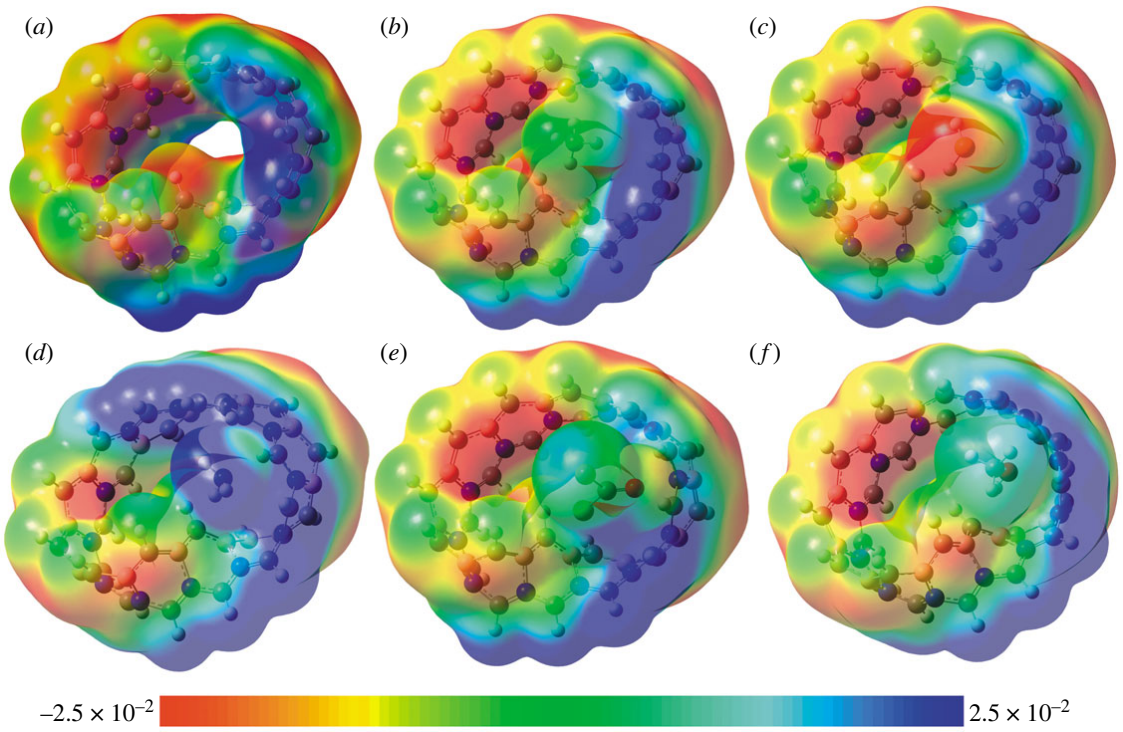
ESP maps of (*a*) MBCN, (*b*) MBCN + CH_4_, (*c*) MBCN + H_2_S, (*d*) MBCN + NH_3_, (*e*) MBCN + COCl_2_ and (*f*) MBCN + CH_3_OH complexes.

The molecular ESP map reveals the asymmetric charge distribution, which shows the variation of ESP in various regions of the structures. The colour scheme for the MEP surface changes from red to blue to signify the electron-rich electrophilic attack zone to the electron-deficient nucleophilic attack zone, respectively [27]. The green region is almost neutral. From figure 7, it is seen that the carbon atom sites bonded with N atoms show positive potential since N is more electronegative than carbon, making C atoms partially positive. Again due to the high electronegativity of C atoms compared to B atoms, the C atom site bonded with B atoms gains more negative potential. After adsorption, the MBCN ESP remains almost similar; however, the adsorbed gas possesses different potentials. CH_4_ shows almost neutrality, representing very much less electrostatic interaction with MBCN. Due to the asymmetric distribution of charges, a net dipole moment (DM) arises in the structures, which determines the polarity of the structure. The DMs of the gas molecules CH_4_, H_2_S, NH_3_, COCl_2_ and CH_3_OH are 0.0 Debye, 1.43 Debye, 1.91 Debye, 1.04 Debye and 1.69 Debye. By contrast, the DMs for MBCN, MBCN + CH_4_, MBCN + H_2_S, MBCN + NH_3_, MBCN + COCl_2_ and MBCN + CH_3_OH are 6.928 Debye, 6.943 Debye, 7.498 Debye, 7.883 Debye, 7.036 Debye and 7.489 Debye, respectively. The DM can vary due to the charge displacement during the adsorption process as well as due to the dipole–dipole interaction between MBCN and the gas molecules. Higher DM means strong interaction between adsorbate and adsorbent. For all gases, the total DM increases after gas adsorption, i.e. the charge distribution becomes more asymmetric, representing the increase of the structures' polarity after gas adsorption. The MBCN structure possesses a very high DM compared to nanosheets [35].

**Figure 8.**
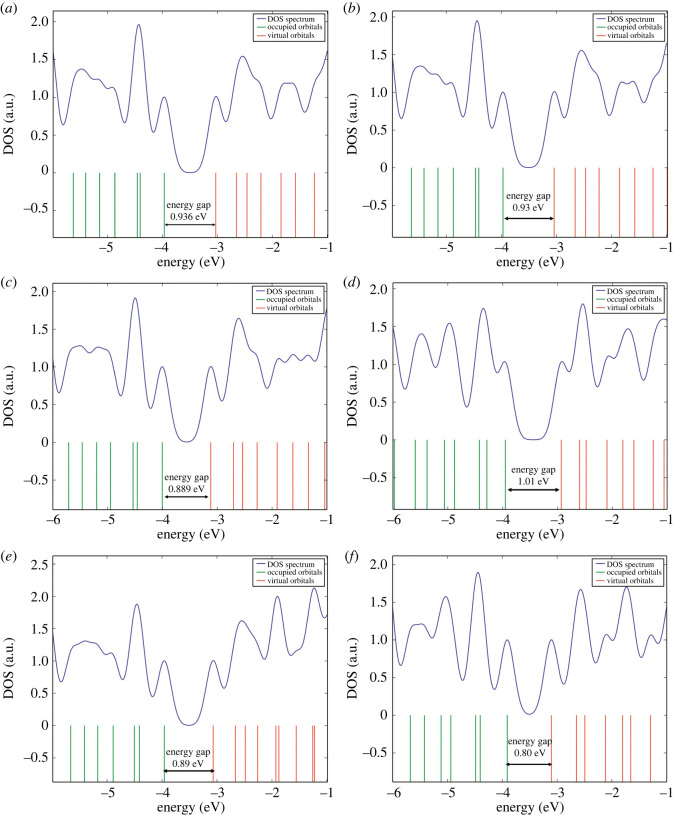
DOS spectra of (*a*) MBCN, (*b*) MBCN + CH_4_, (*c*) MBCN + H_2_S, (*d*) MBCN + NH_3_, (*e*) MBCN + COCl_2_ and (*f*) MBCN + CH_3_OH complexes.

The DOS spectra were studied for all the complexes to better understand electronic variations (figure 8). The HOMO and LUMO energy states of the pristine MBCN varied very slightly after gas adsorption. The energy gap has been obtained from the difference of HOMO and LUMO of the complexes. For pristine MBCN, the energy gap is 0.936 eV, which shows slight variation after gas adsorption. After adsorption of NH_3_ gas, the energy gap slightly increased to 1.01 eV, whereas for other gases, the energy gap decreased slightly. The variation of the energy gap suggests the alteration of electronic states due to the adsorption of the gas molecules. For CH_4_ adsorption, the energy gap variation is minor, which indicates that CH_4_ caused a very weak interaction with MBCN. The HOMO and LUMO energy states of the pristine MBCN varied very slightly after gas adsorption.

The conductivity (*σ*) is related to the energy gap by the Arrhenius equation [19]:

3.2 $$\sigma \propto e^{-E_{g}/2KT},$$

where *E*_g_, *K*, and *T* are the energy gap, Boltzmann constant, and temperature, respectively. The conductivity is exponentially related to the energy gap of the complexes, i.e. the conductivity decreases with increasing energy gap and vice versa. Since the MBCN + NH_3_ complex possesses the maximum energy gap in this research, the complex will show the least conductivity.

In comparison, maximum conductivity is observed for MBCN + CH_3_OH. According to the equation, the conductivity of the structures increases with temperature due to the negative temperature coefficient of resistance. The conductivity of the structures follows the following trend: *σ*(MBCN + NH_3_) < *σ*(MBCN) < *σ*(MBCN + CH_4_) < *σ*(MBCN + COCl_2_) < *σ*(MBCN + H_2_S) < *σ*(MBCN + CH_3_OH).

**Figure 9.**
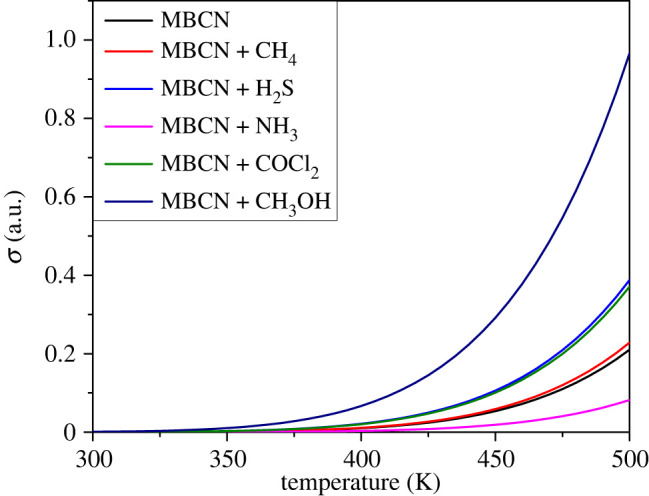
Conductivity variation with temperature.

Figure 9 shows the variation of conductivity of MBCN and MBCN + gas complexes with temperature obtained from equation (3.2). The observed conductivity is maximum for MBCN + CH_3_OH whereas it is minimum for MBCN + NH_3_ complex. Due to the semiconducting nature the conductivity of all structures increases with temperature.

### 3.4. Optical properties

**Figure 10.**
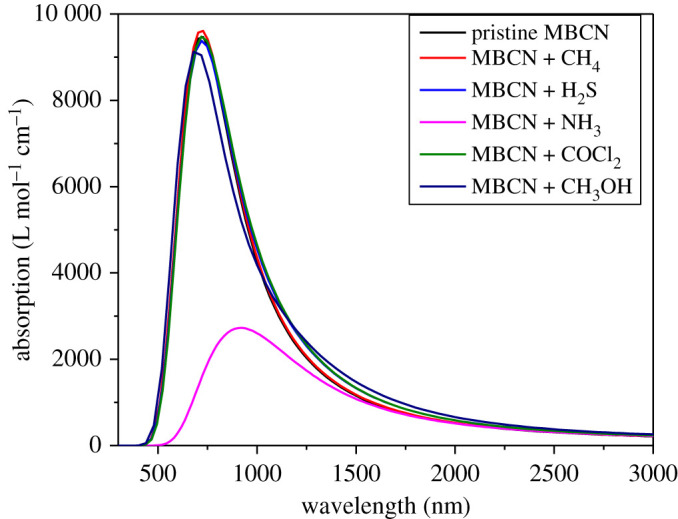
Molar absorption coefficient of MBCN and MBCN + gas complexes.

All the complexes show a high absorption coefficient of order 10^4^ cm^−1^ in the longer wavelength region of the visible spectrum (figure 10), suggesting MBCN as a potential material in various optoelectronic applications. The absorption coefficient of MBCN slightly varied after adsorption of CH_4_, H_2_S, COCl_2_ and CH_3_OH molecules, whereas it decreased significantly after NH_3_ adsorption. Since the optical conductivity, an essential property to describe optoelectronic performance, is proportional to the absorption coefficient, it signifies that the conductivity has decreased by a great extent after NH_3_ adsorption.

### 3.5. Quantum molecular descriptors

QMD investigations are necessary to decode the information on the reactivity and chemical stability of the adsorbent and its adsorbates. The global hardness (*η*) can be thought of as a barrier to charge transfer, with a greater value indicating lesser chemical reactivity and more chemical stability, which is the opposite of global softness. The global hardness of MBCN is reduced after the adsorption of CH_4_, H_2_S, COCl_2_ and CH_3_OH gases, whereas it increases after the adsorption of NH_3_ gas (table 6). This represents that the MBCN + NH_3_ complex opposes charge transfer, i.e. the conductivity decreases. The slightest variation in hardness is observed after CH_4_ adsorption suggesting that the MBCN structure very weakly interacts with CH_4_ molecules. The value of chemical potential, electrophilicity and electronegativity decreased significantly after the adsorption of NH_3_ gas, whereas it increased after adsorption of the other toxic gases.

**Table 6.** Global hardness, softness, chemical potential, electrophilicity and electronegativity of the complexes.

structures	hardness (eV)	chemical potential (eV)	global softness (eV^−1^)	electrophilicity (eV)	electronegativity (eV)
MBCN	0.469	−3.498	2.133	13.051	3.498
MBCN + CH_4_	0.465	−3.507	2.149	13.218	3.507
MBCN + H_2_S	0.442	−3.56	2.26	14.322	3.56
MBCN + NH_3_	0.51	−3.442	1.963	11.623	3.442
MBCN + COCl_2_	0.444	−3.515	2.25	13.899	3.515
MBCN + CH_3_OH	0.403	−3.507	2.481	15.255	3.507

### 3.6. Thermodynamic properties

The studied complex structures’ thermodynamic properties were also examined at temperature of 298.15 K and pressure of 1 atm to confirm their thermal stability. During the chemical reaction, the change in enthalpy allows us to determine if a reaction is endothermic (Δ*H* > 0) or exothermic (Δ*H* < 0), and the change in Gibbs free energy represents whether the gas molecules and MBCN have a spontaneous interaction (Δ*G* < 0) or not (Δ*G* > 0). The change in entropy (Δ*S*) is also studied, which can be either negative or positive. The negative change represents that the structure is thermodynamically more stable. The negative values of Δ*H* for all complexes suggested that all adsorption processes are exothermic. The adsorption of NH_3_ and CH_3_OH is spontaneous, whereas the adsorption of the other three gases is nonspontaneous (table 7). Since Δ*S* < 0 for all the complexes, all the reactions are thermodynamically ordered.

**Table 7.** Thermodynamic parameters of the complexes.

structures	Δ*H* (eV atom^−1^)	Δ*G* (eV atom^−1^)	Δ*S* (eV K^−1^)
MBCN + CH_4_	−0.059	0.256	−0.0011
MBCN + H_2_S	−0.234	0.219	−0.0015
MBCN + NH_3_	−0.555	−0.025	−0.0018
MBCN + COCl_2_	−0.198	0.263	−0.0015
MBCN + CH_3_OH	−0.526	−0.032	−0.0017

## 4. Conclusion

The MBCN structure has been modelled and successfully optimized using DFT. The real frequencies of the molecular vibrations revealed that the structure could exist naturally. The structure showed semiconducting nature with a 0.936 eV energy gap, high absorption coefficient and a strong DM. Due to the high absorption coefficient, MBCN is also a potential material for optoelectronic research. The structure showed good sensitivity towards CH_4_, H_2_S, NH_3_, COCl_2_ and CH_3_OH gas molecules. Methanol showed the maximum interaction with the adsorbent (recovery time approx. 8 × 10^−3^ s), whereas CH_4_ showed the minimum interaction (recovery time approx. 7 × 10^−12^ s). The HOMO and LUMO energies, DOS, energy gap and optical absorbance altered significantly after gas adsorption. The QMD studies revealed better sensitivity of the MBCN structure towards the gas molecules. The thermodynamic properties verify that all the adsorption reactions are exothermic with better thermodynamic stability.

## Data Availability

Gaussian input files are available from the Dryad Digital Repository: https://doi.org/10.5061/dryad.w9ghx3frv [36].
